# Ammonia as a Potential Neurotoxic Factor in Alzheimer's Disease

**DOI:** 10.3389/fnmol.2016.00057

**Published:** 2016-08-08

**Authors:** Aida Adlimoghaddam, Mohammad G. Sabbir, Benedict C. Albensi

**Affiliations:** ^1^Division of Neurodegenerative Disorders, St. Boniface Hospital ResearchWinnipeg, MB, Canada; ^2^Department of Pharmacology & Therapeutics, University of ManitobaWinnipeg, MB, Canada

**Keywords:** ammonia, ammonia transporters, toxicity, Alzheimer disease, energy metabolism, mitochondrial dysfunction, glutamatergic, GABAergic

## Abstract

Ammonia is known to be a potent neurotoxin that causes severe negative effects on the central nervous system. Excessive ammonia levels have been detected in the brain of patients with neurological disorders such as Alzheimer disease (AD). Therefore, ammonia could be a factor contributing to the progression of AD. In this review, we provide an introduction to the toxicity of ammonia and putative ammonia transport proteins. We also hypothesize how ammonia may be linked to AD. Additionally, we discuss the evidence that support the hypothesis that ammonia is a key factor contributing to AD progression. Lastly, we summarize the old and new experimental evidence that focuses on energy metabolism, mitochondrial function, inflammatory responses, excitatory glutamatergic, and GABAergic neurotransmission, and memory in support of our ammonia-related hypotheses of AD.

## Introduction

All living organisms produce ammonia as a byproduct of cellular metabolism. At high concentrations, ammonia is toxic and causes deleterious effects to the cell (Cooper and Plum, 1987). Effects include disruption of cellular energy metabolism, mitochondrial dysfunction, modulation of inflammatory responses, and neurotransmission in neurons. Existing evidence suggests that accumulation of ammonia in the brain affects neuronal function and may lead to several neurological abnormalities. Therefore, ammonia could be a causative factor for Alzheimer disease (AD) and may be involved in the progression of the disease. In 1993, Seiler for the first time published his hypothesis about a linkage between ammonia and AD (Seiler, 1993). However, since then, few research undertakings have directly shown a pathophysiological role of ammonia within the AD brain. In this review, toxicity and transport of various forms of ammonia are briefly described. AD–related factors are also highlighted and then built upon to discuss the contribution of ammonia to AD.

## Sources of brain ammonia

**Figure 1:**
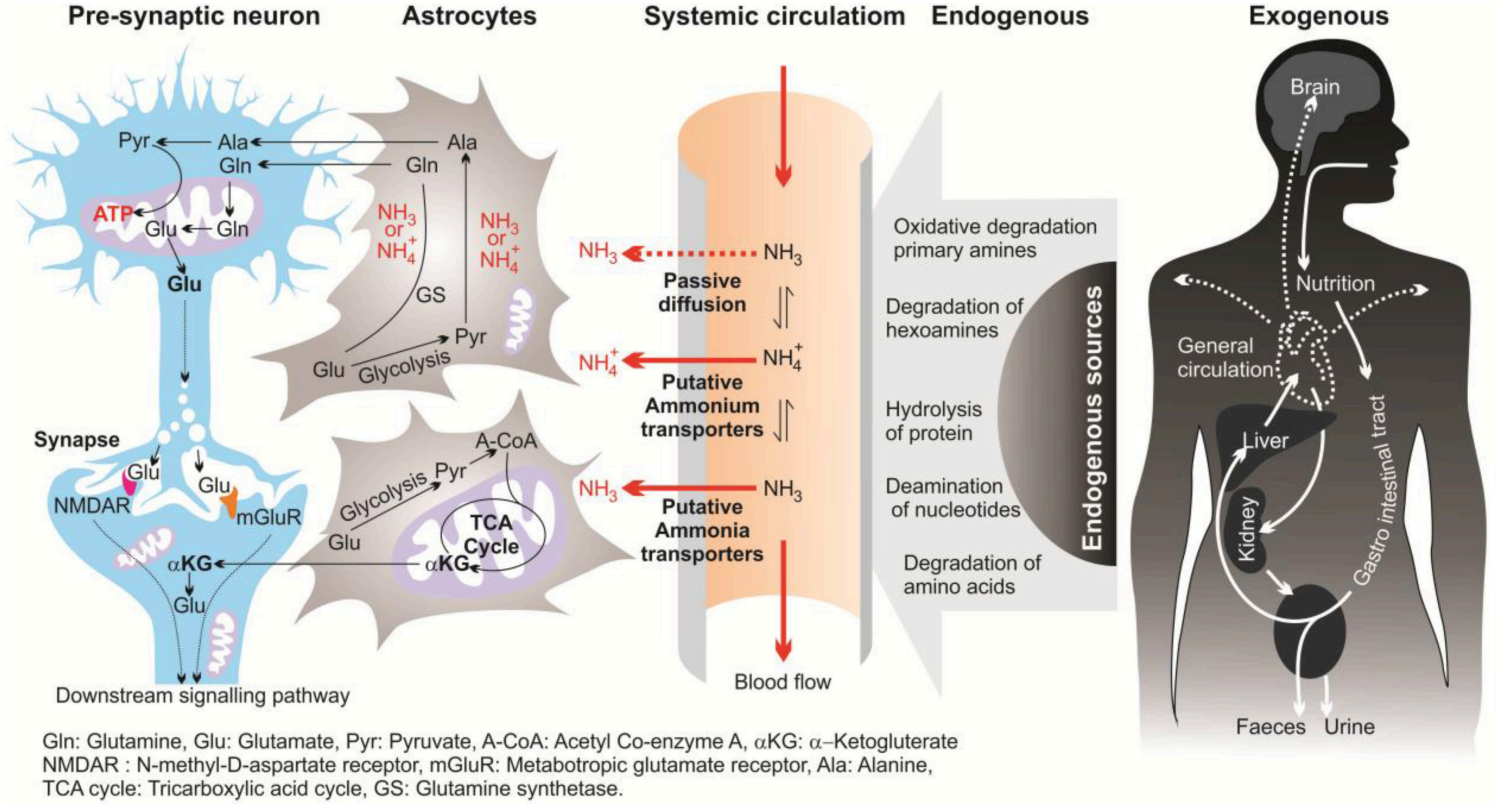
**Diagrammatic representation of sources, transport and metabolism of ammonia in the brain**.

In this review, the term “ammonia” refers to two chemical species (NH${}_{4}^{+}$ and NH_3_) and when referring to a specific molecular form, “NH${}_{4}^{+}$” or “NH_3_” will be used. In mammalian brains, ammonia is derived mostly from the metabolism of the putative neurotransmitters glutamate and aspartate, and monoamines. In the brain, ammonia derives from two main pathways; endogenous and exogenous sources (Figure 1; Seiler, 1993, 2002; O'Donnell, 1997). Endogenous sources of brain ammonia involve: hydrolysis of proteins; degradation of amino acids (e.g., glutamine, asparagine, and glycine) and degradation of hexamines; deamination of amino-purines, amino-pyrimidines, and oxidative deamination of primary amines. One endogenous source comes from abnormalities in glucose metabolism which results in excessive ammonia concentrations within the cerebral cortex (Hoyer et al., 1988). Aside from liver dysfunction, ammonia also could be generated from the deficiency of brain metabolism or detoxification processes resulting from the major reduction in the activity of glutamine synthesis (Suarez et al., 2002). Another source of brain ammonia is adenosine-3-monophosphate (AMP) deaminase, which regulates the purine nucleotides and converts AMP to inosine monophosphate and ammonia. In 1998, Sims and colleagues found that the activity of adenosine-3-monophosphate (AMP) deaminase is approximately 2-folds greater in AD brains compared with control individuals (Sims et al., 1998). These outcomes led to the assumption that over-activity of AMP deaminase could be a source of elevated ammonia levels during deficient glucose metabolism in AD (Sims et al., 1998). Further, monoamine oxidase (MAO) could be involved, to a lower extent, in the process of ammonia production due to degradation of neurotransmitters and non-transmitter monoamines.

**Figure 2:**
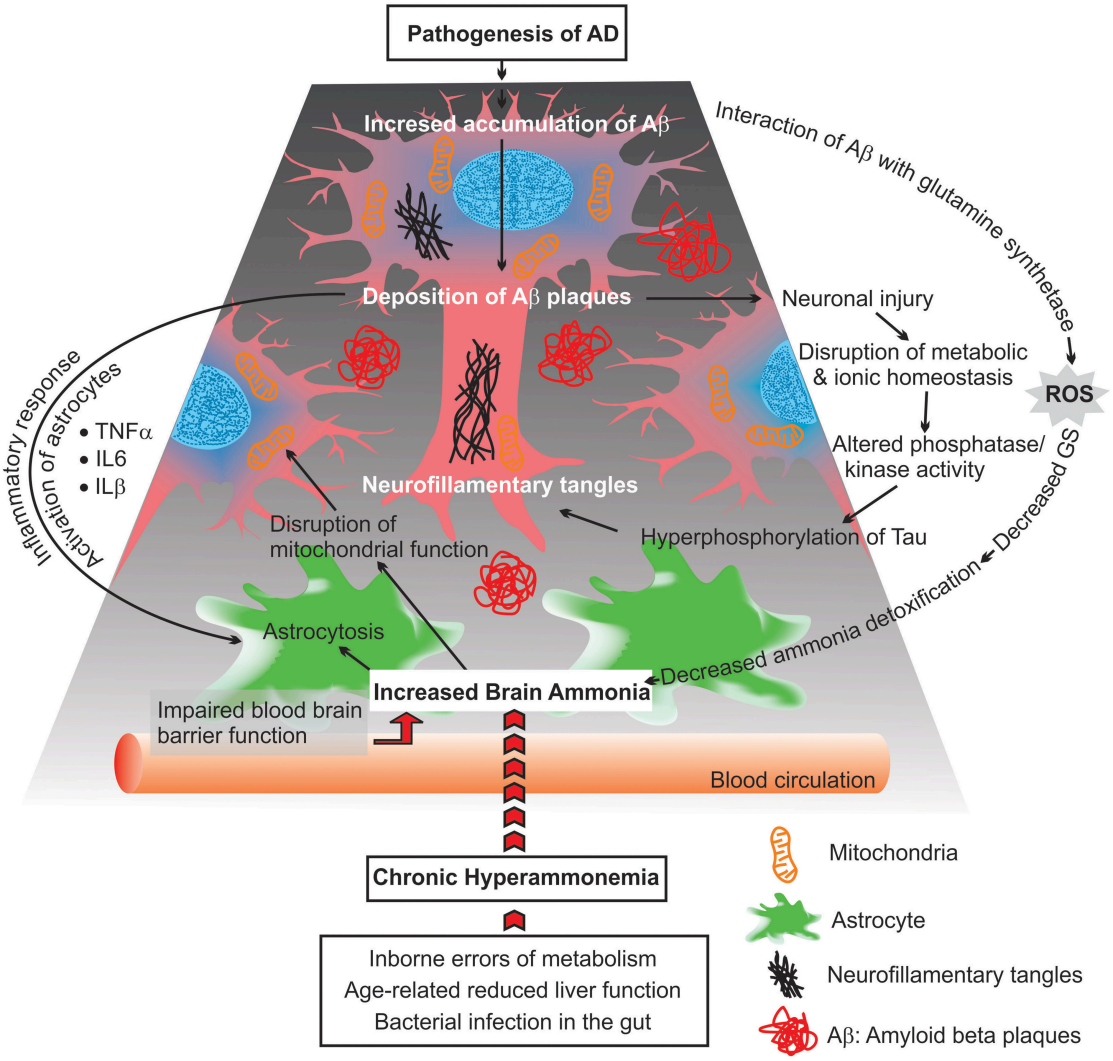
**Scheme diagram representing possible consequences of chronic hyperammonemia, presumed to lead to progressive impairment of astrocytes and neuronal damage as well as mitochondrial malfunction**.

Exogenous sources produce large quantities of ammonia in the gastrointestinal tract, resulting from bacterial degradation of urea and deamination of amino acids (Marcaggi and Coles, 2001). Urea cycle failure and deficient hepatic urea formation, inborn errors of metabolism, bacterial infection in the gut are major causes of accumulation of ammonia in the brain (Figure 2). Evidence to date indicates that ammonia is a key pathogenetic factor of hepatic encephalopathy (HE) and a major neurotropic factor of liver failure (Häussinger and Schliess, 2008; Lemberg and Fernandez, 2009). Additionally, some studies suggest that excessive ammonia levels in mammals have been related to AD due to toxic accumulation of glutamine in astrocytes, what leads to cell swelling and finally cell death (Butterworth, 2002). However, evidence for a role for ammonia in the pathology of AD is still not concrete.

## Toxicity of ammonia

Ammonia is the major end product of cellular amino acid metabolism (Wright, 1995). Ammonia is a highly toxic material in animals at even sub-millimolar concentrations (Marcaida et al., 1992; Britto and Kronzucker, 2002). Ammonia is a weak base with a pK of 9.2–9.8, depending on the temperature and salinity of the media (Cameron and Heisler, 1983). In body fluids with a physiological pH (~7.4) the major fraction of ammonia (*ca.* 99%) appears as NH${}_{4}^{+}$ and the rest appears as NH_3_ (Figure 1). Both forms of ammonia, NH_3_ and NH${}_{4}^{+}$, have toxic effects by potentially disturbing the pH balance of the cytoplasm and body fluids (Erickson, 1985). Due to the small size and uncharged state, NH_3_, can diffuse down its partial pressure gradient (Δ*P*NH_3_) across lipid bilayers into acidic vesicles such as lysosomes and impair the appropriate function of Golgi vesicles and lysosomal proteases. This occur since NH_3_ can alter the intraorganelle pH away from the optimal necessary pH for normal operation (Seglen, 1983). Ammonia formed from glutamate deamination, where its toxicity results from disruption of the H^+^ gradient across the inner membranes of mitochondria. Due to its relative alkalinity, the mitochondrial pH as compared to the cytoplasmic pH results in an outwardly directed Δ*P*NH_3_ from the matrix to the intermitochondrial space. Thus, NH_3_ exits the mitochondrial matrix along this gradient and binds to H^+^ in the inter-membrane space, thereby eliminating the H^+^ gradient necessary for ATP synthesis (Cooper and Plum, 1987). Therefore, a dropping pH drives oxidative phosphorylation where ammonia is acting as an H^+^-gradient-uncoupler (O'Donnell, 1997). In addition, hydrated NH${}_{4}^{+}$ and K^+^ ions have the same ionic radius of 1.45 Å (Knepper et al., 1989; Weiner and Hamm, 2007), which could result in competition at the K^+^ binding site of K^+^-channels. This competition affects neuronal excitability and membrane potential in mammalian neurons (Cooper and Plum, 1987). It has also been demonstrated that high ammonia concentrations can depolarize hippocampal neurons (Bosoi and Rose, 2009). Elevated ammonia also causes major damage in the CNS, including changes in blood-brain barrier (BBB) morphology (Laursen and Diemer, 1979), modification in astrocyte and neuron morphology (Gregorios et al., 1985), and HE (Butterworth, 2002).

In addition, elevated ammonia levels in mammals have been related to AD due to toxic accumulation of glutamine in astrocytes, which leads to cell swelling and ultimately cell death (Butterworth, 2002). In microglia and astroglioma cell-lines, ammonia affects major functional activities such as phagocytosis and endocytosis. In addition, ammonia modifies the secretion of cytokines and elevates the activity of lysosomal hydrolases (Atanassov et al., 1994, 1995). Further, ammonium ions inhibit important enzymes involved in protein metabolism, such as alpha-ketoglutarate dehydrogenase and isocitrate dehydrogenase, which ultimately leads to free radical generation (Cooper and Plum, 1987). Moreover, elevated ammonia concentration reduces the activity of the antioxidant enzymes, and results in inhibition of mitochondrial electron transport chain (ETC) (Murthy et al., 2001). In rat brain it has been shown that high ammonia concentrations interact with mitochondria and inhibit complexes I–IV of the ETC (Veauvy et al., 2002). Marcaida and coworkers found evidence that ammonia toxicity is mediated by excessive activation of *N*-methyl-D-aspartate (NMDA)-type glutamate receptors in the brain. As a consequence, cerebral ATP is depleted while intracellular Ca^2+^ increases with subsequent increases in extracellular K^+^, leading to cell death (Marcaida et al., 1992). Additionally, neurotoxicity is mediated by a direct inhibitory effect of ammonia on the astrocytic EAAT-1 (GLAST) and EAAT-2 (GLT-1) transporters, which are responsible for the removal of glutamate from the neuronal synapse (Knecht et al., 1997; Norenberg et al., 1997; Chan et al., 2000). In most species, including mammals, the ammonia concentration of body fluids is typically low (*ca.* 50–250 μM) (Cooper and Plum, 1987). Concentrations exceeding 1 mM are usually toxic to mammalian cells (Hrnjez et al., 1999). Because of its toxicity an effective ammonia detoxification or excretion system is crucial to maintain cellular and body fluid ammonia levels within a tolerable range to ensure normal systemic functions.

## Ammonia transporters

### Rhesus proteins (Rh)

To protect the brain from ammonia-induced stress, understanding the specific role of ammonia transporters, which are putatively involved in the ammonia transport system, is critical. The ammonia-transporting proteins in humans are the Rhesus (Rh) proteins: RhAG, RhBG, and RhCG. It has been shown that total ammonia levels in erythrocytes are greater than three times as compared to plasma ammonia levels (Huizenga et al., 1994). The RhAG (erythroid- Rh) complex may play a role in keeping the total blood ammonia level low by transporting ammonia inside the red blood cells (RBCs) (Huang et al., 2004). In mammals, RhAG is located in erythrocytes and erythropoietic tissues (Nakada et al., 2007). The RhBG and RhCG (Non-erythroid Rh) proteins have been distributed in various organs such as brain, kidney, liver, and skin, more specifically in locations where ammonia production and excretion is crucial (Liu et al., 2000; Weiner and Verlander, 2003). Gene expression of the Rh proteins from the brain of rainbow trout, Oncorhynchus mykiss, was significantly up-regulated upon ammonia-induced stress (Nawata and Wood, 2009). This suggests that the brain Rh proteins contribute at least partially to the ammonia excretion process.

Functional expression studies of vertebrate Rh proteins differ and are not clear-cut as to the exact molecular species (NH_3_ or NH${}_{4}^{+}$) that is transported. When expressed in *HeLa* cells human erythroid RhAG appears to transport both types of species (Benjelloun et al., 2005). Tracer studies proposed that NH${}_{4}^{+}$ is transported across the BBB, from plasma to brain, via Rh proteins (Ott and Larsen, 2004). The physiological role of human Rh- proteins was revealed by expressing RhCG in yeast strains deficient in endogenous ammonia transporters (triple-MepΔ; lacking all three ammonium transporters). Growth of triple-mepΔ cells expressing human Rh on a medium, where ammonia is the only source of nitrogen showed that RhCG is capable of transporting ammonium in yeast cells (Marini et al., 2000). However, the debate regarding transport specificity of members of the Rh family is ongoing. Recently, the X-ray crystallographic analysis on RhCG revealed that monomers of Rh proteins contain a hydrophobic pore element, while the protein form in a trimeric complex promotes the passage of gas form of ammonia (NH_3_) (Gruswitz et al., 2010). Furthermore, topological analyses indicated that the structures of the 12 transmembrane (TM) domains are conserved in all Rh proteins (Huang and Peng, 2005). Sequence alignment analyses of Rh proteins among mammals, fish, crustaceans, nematodes and insects suggested that Rh proteins are phylogenetically related and most likely share a conserved ammonia transport function (Weihrauch et al., 2004; Huang and Peng, 2005; Zidi-Yahiaoui et al., 2009; Adlimoghaddam et al., 2016).

### Aquaporins (AQP)

Aquaporins (AQPs) are membrane proteins that operate as channels for the transport of water. Some members of the AQP family of proteins can also be permeable to other molecules such as glycerol, NH_3_, urea, NO, O_2_, CO_2_, H_2_O_2_, and As(OH)_3_. Ammonia transport capabilities were confirmed for four members of the mammalian aquaporin family, AQP3, AQP7, AQP8, and AQP9, when expressed in *Xenopus* oocytes (Saparov et al., 2007; Litman et al., 2009). The gene expression analysis showed that the expression of AQP-4 is downregulated in the astrocytes of Spf/GFAP-EGEP mice. However, in a rat model of acute liver failure, the protein expression level of AQP-4 significantly increased, which appeared to precede the onset of astrocyte swelling. Therefore, astrocytes may respond to elevated blood ammonia concentrations by an alteration in expression levels of AQP-4 (Rao et al., 2010). Moreover, it was shown that knocking out the AQP-4 gene in cultured astrocytes is capable of preventing ammonia-induced cell swelling (Rama Rao et al., 2014).

### V-type H^+^-ATPase (V-ATPase)

Another way to transport ammonia occurs via vacuolar-type H^+^-ATPase (V-ATPase) (Weihrauch et al., 2002). Although the transporter itself is not directly involved in ammonia transport, pumping of protons via V-ATPase to the outside of the epithelium (dropping pH) generates an outwardly directed Δ*P*NH_3_ which facilitates NH_3_ excretion across the membrane either via passive membrane diffusion or potentially via NH_3_ permeable channels, such as Rhesus proteins (Nawata et al., 2007; Musa-Aziz et al., 2009; Gruswitz et al., 2010). This transporter localized at high expression levels in brain tissues, which may reveal a particular role in neural tissues beyond its housekeeping roles. For example, *in vitro* ammonia treatment stimulates the activity of H^+^-ATPase in synaptic vesicles of the rat brain (Albrecht et al., 1994).

### Na^+^/H^+^ exchangers (NHE)

Na^+^/H^+^ exchangers (NHE) isoforms are widely distributed in the mammalian CNS. This in turn is leading to the movement of Na^+^ down its concentration gradient into the cytosol through plasma membrane localized NHEs in exchange for H^+^. All cells actively regulate their intracellular pH and NHE is potentially involved in acid-base regulation. For example, NHE-1 is highly expressed in neurons and astrocytes to contribute in cellular pH regulation and cell volume (Pizzonia et al., 1996; Yao et al., 1999; Chesler, 2003). In addition to cellular pH regulation, NHEs would promote an acidification across lipid bilayers and thereby assist ammonia trapping as suggested in the proximal tubule (Hamm and Simon, 1990). However, whether transporting protons could assist ammonia trapping for mammalian astrocytes is not completely understood.

### Transport of NH${}_{4}^{+}$

The ionic form of ammonia (NH${}_{4}^{+}$) cannot diffuse along biological membranes; however, they can permeate epithelia across an electrochemical gradient via the paracellular pathway based on the ion permeability of the tight junctions. Moreover, since hydrated NH${}_{4}^{+}$ and K^+^ have a similar size and ionic radius (Knepper et al., 1989; Weiner and Hamm, 2007), NH${}_{4}^{+}$ to a certain extent can compete with K^+^ and replace K^+^ as a substrate in K^+^ transporting proteins, such as Na^+^/K^+^-ATPase (NKA), K^+^-channels, and Na^+^/K^+^/2Cl^−^ co-transporters (NKCC) (Marcaggi and Coles, 2001; Weiner and Hamm, 2007; Larsen et al., 2014; Adlimoghaddam et al., 2015; Hertz et al., 2015).

### Na^+^/K^+^-ATPase (NKA)

Basolaterally localized Na^+^/K^+^-ATPase (NKA), hydrolyzes ATP to pump three Na^+^ ions from the cytosol out of the cell while concurrently pumping two K^+^ into the cell (Skou, 1957). The NKA creates an electrochemical gradient of Na^+^ and also generates a negative membrane potential that is critical for many transepithelial transport processes. These processes are in favor of maintaining cellular osmolality and energizing various sodium dependent transporters such as NKA (Hu and Kaplan, 2000; Kaplan, 2002). The involvement of the NKA in ammonia transport processes has been shown in many species and various tissues, including the mammalian astrocytes. In addition, enzyme activity measurements from rat astrocyte cultures revealed that the NKA also accepts NH${}_{4}^{+}$ as a substrate substituting K^+^ and is thereby directly involved in the active transport of NH${}_{4}^{+}$ (i.e., from the body fluids into the cytoplasm; Chan et al., 2013; Rangroo Thrane et al., 2013). Protein and mRNA expression analyses from ammonia-induced astrocyte cultures indicated that NKA was up-regulated in response to high ammonia concentrations, suggesting an important role of NKA in an ammonia transport mechanism (Xue et al., 2010). Additionally, blocking NKA by using a ouabain inhibitor leads to reduce ammonium-induced astrocytic swelling, suggesting NKA is involved in ammonia homeostasis and cell swelling (Dai et al., 2013; Song and Du, 2014).

### K^+^-channels

K^+^-channels, due to their ubiquitous cellular presence, are likely one of the key candidates to mediate transmembrane NH${}_{4}^{+}$ transport. In accordance with the aforementioned competition between K^+^ and NH${}_{4}^{+}$, it has been suggested that NH${}_{4}^{+}$ can permeate through the BBB with the possible participation of barium-inhibitable K^+^ channel (Ott and Larsen, 2004). Additionally, it has been demonstrated that in cultured astrocytes, inward-rectifying K^+^ channel genes (*Kir4.1* and *Kir5.1*) significantly downregulated in conditions of hyperammonemia (Lichter-Konecki et al., 2008). These results suggest that alteration of K^+^ channels could either reveal a protective response by astrocytes to elevated blood NH${}_{4}^{+}$ levels, or it is responsive to increased extracellular brain K^+^ and plasma K^+^ concentration. Thus, alteration in brain K^+^ level could have a key impact on neuronal activity and network activity during and after hyperammonemia. More studies will be needed to investigate details regarding the mechanisms involved in the transport of ammonia through K^+^ channel inill be needed to investigate details brain.

### Na^+^/K^+^/2Cl^−^ co-transporter (NKCC)

The basolaterally or apically localized Na^+^/K^+^/2Cl^−^ co-transporter (NKCC) transports Na^+^, K^+^, and 2Cl^−^ in an electroneutral manner. Two isoforms of NKCC (1 and 2) have been identified in several cells and tissues. In mammals, NKCC1 is located in many cell types such as astrocytes and neurons, while NKCC2 is presented mostly in the kidney.

Recent studies indicated that NKCC1 in mammalian brain tissue and in brain cell cultures accept NH${}_{4}^{+}$ as a substrate, subtitling K^+^, which demonstrates the importance of NKCC in the ammonia transport system in astrocytes. The NKCC1 has been shown to transport NH${}_{4}^{+}$ in isolated astrocytes (Jayakumar et al., 2008). Recent studies have also shown that in cultured astrocyte from rats, the NKCC1 was activated in response to NH${}_{4}^{+}$ exposure. Thus, increasing NKCC activation was associated with astrocyte swelling, a process that was blocked by a NKCC inhibitor (Jayakumar et al., 2008; Rangroo Thrane et al., 2013). These studies highlight the role of NKCC in ammonia homeostasis and astrocyte swelling.

## The necessities of comparative studies and nitrogen transport

Overall, dysregulation of the nitrogen transport system due to ammonia toxicity and any changes in ammonia transporter expression and function could affect brain ammonia homeostasis and function, which may lead to severe neuronal damage in the AD brain. As mentioned above, changes in the expression of ammonia transporters most likely play a critical role in ammonia homeostasis and cell swelling; however, a possible link between the altered ammonia transporter function and AD is still missing. Therefore, more studies will be needed to investigate details regarding the mechanisms involved in the transport of toxic ammonia in the AD vs. normal brain. Elucidation of clinical pathological mechanisms related to the ammonia transport system may provide common links to the etiology of AD. Together these insights are crucial for developing therapeutic drugs to modify dangerous ammonia influxes that cause elevated systemic ammonia levels and eventually lethal brain damage in AD.

## Alzheimer's disease (AD)

Currently, AD is the most common progressive neurodegenerative disease in the world (Sperling et al., 2011). It is clinically characterized by disruption to synaptic plasticity, learning, memory, and several other cognitive functions (Albert, 1996). Neuropat hologically, AD is characterized by the development of intracellular neurofibrillary tangles (NFT) formed from the composition of hyperphosphorylated tau protein and accumulations of extracellular senile plaques (SP) that aggregate from the deposition of amyloid-β (Aβ) (Price and Morris, 1999; Sperling et al., 2011). Another histological hallmark of the disease is the unfavorable metabolism of amyloid-β precursor protein (AβPP) in SP and the subsequent accumulation of AβPP in damaged axons. The overexpression of AβPP generates a cascade of events that include hyperphosphorylated tau that leads to synaptic failure (Ward et al., 2012).

Besides tau hyperphosphorylation (Grundke-Iqbal et al., 1986) and Aβ deposition (Hardy and Selkoe, 2002), other pathological aberrations include: transcriptional dysregulation (Pastorcic and Das, 2007) modified neuroinflammatory process (Granic et al., 2009) and astrogliosis (Akude et al., 2011). The etiology and neuropathogenesis of AD suggest that this disease is complex and is better thought of as a multifactorial neurodegenerative disorder involving various proteins (Carreiras et al., 2013). Regarding causative factors in AD, various hypotheses have been proposed including impaired energy metabolism, mitochondrial dysfunction (Hoyer, 1998), alterations in neurotransmitter receptors systems (such as GABA, glutamate, MAO; Sims et al., 1998; Jones, 2002), micro-RNA deficiency (Nixon, 2013), cell cycle re-entry (Bonda et al., 2010), cholinergic deficiency (Pinto et al., 2011), neuroimmunomodulation (Akude et al., 2011), and deficiency in calcium homeostasis (Berridge, 2010) to name a few.

Among the neurotoxic agents that have been studied in relation to the pathology of AD, the effect of ammonia, as a potent neurotoxin, has received less attention than it deserves. In this review several hypotheses are mentioned regarding the etiology of ammonia in AD including deficiency in glycose metabolism, mitochondrial dysfunction, impairment of glut amatergic and GABAergic neurotransmission, dysregulation of inflammatory responses, and memory dysfunction.

## Impaired energy metabolism and mitochondria in AD and hyperammonia conditions

Glucose is the main source of energy within the brain and dysfunction of glucose metabolism has critical pathophysiological consequences. Several studies indicate a significant reduction in glycolytic process in brains with dementia (Meier-Ruge et al., 1994; Simpson et al., 1994; Hoyer, 2000, 2004). The dysregulation of glucose metabolism has been demonstrated by comparing the enzymatic activity of glucose transporters (Simpson and Davies, 1994), hexokinase (Marcus and Freedman, 1997), pyruvate dehydrogenase (PDH) (Bubber et al., 2005) and enzymes of the tricarboxylic acid (TCA) cycle in AD vs. control individuals (Kosenko et al., 2014).

High ammonia concentrations lead to elevated content of astrocytic glutamine with a decrease in glutamate concentration which causes reduction in the activity of the malate-asparate shuttle (MAS). As a result of impaired MAS, the pyruvate/lactate ratio decreases in astrocytes. Unrelated to MAS activity, high ammonia concentrations in both astrocytes and neurons can inhibit decarboxylation of alpha-ketoglutarate in the TCA cycle, which leads to inhibition of PDH (Hertz and Kala, 2007).

Beside deficiency in glucose metabolism, the functionality of mitochondria is affected in AD brains. This includes: increases in reactive oxygen species (ROS) production, disruption in the balance between mitochondrial fission and fusion, changes in mitochondria morphology, mitochondrial enzymatic failure, and a reduced rate of mitochondrial axonal transport (Figure 2; Zhu et al., 2013; Cadonic et al., 2015).

Although, it has been hypothesized that mitochondrial regulation is generally genetically inherent, the activity of mitochondria could be influenced by other neurotoxic factors such as ammonia. Several studies indicate that ammonia compromises various parts of the cellular bioenergetic machinery. For example, the activity of several ETC enzymes, mitochondrial cytochrome c oxidase, glutathione peroxidase, and superoxidase dismutase are significantly reduced in ammonia-treated brain (Kosenko et al., 1997, 1999, 2004, 2007; Qureshi et al., 1998; Esteves et al., 2009). Also, activity of superoxidase, ROS and Poly (ADP-Ribose) polymerase (PARP) increased in brain mitochondria upon anammonia-induced stress condition (Kosenko et al., 2003, 2004; Moreira et al., 2008). Existing evidence indicates that energy metabolism is compromised in AD and ammonia is involved in the disruption of energy metabolism (i.e., mitochondrial dysfunction) in AD. However, more studies are required to obtain a better understanding of how mitochondria are affected by high ammonia concentrations, how AD vs. normal mammalian brain cells handle energy deficiency, and how these organelles protect themselves from a massive influx of toxic ammonia into the brain.

## Ammonia effects on excitatory glutamatergic and GABAergic neurotransmission

One of the crucial roles of astrocytes is to protect neurons against excitotoxicity by taking up excess ammonia (NH_3_) and glutamate (Glu) and converting it into glutamine (Gln) via adenosine tri-phosphate dependent glutamine synthase (GS). Within the liver and neurons, Gln is hydrolyzed via phosphate-dependent glutaminase to Glu and ammonia (NH_3_) (Zielke et al., 1989; Smith, 1990).

It has been shown in individuals with HE that there is a lack of balance between excitatory and inhibitory neurotransmission. The major inhibition is due to decreased expression of Glu receptors which leads to reduced glutamatergic tone. Moreover, the inhibition of glutamate transporters (Glt-1) in HE patients results in reductions in Glu re-uptake into astrocytes following excessive extrasynaptic accumulation of Glu (Albrecht and Jones, 1999).

Moreover, abnormal ammonia metabolism in AD brains correlated with decreases of astrocytic GS activity (Suarez et al., 2002). Changes in the expression level of GS upon an ammonia-induced stress condition may alter astroglial morphology (astrocytosis), which can reflect on neuronal function (Figure 2). The changes in the regulation of GS suggest that the Glu-Gln cycle may be differentially impaired in AD. Additionally, the lower activity of GS is related with the density of extracellular deposits of Aβ and SP in the cortex of AD brains (Le Prince et al., 1995). It was demonstrated that Aβ can interact with GS and induce oxidative inactivation of this enzyme as well as enhance the neurotoxicity of Aβ. Consistent with these findings, it is suggested that there is a linkage between impaired ammonia detoxification (due to alteration in GS activity) and amyloid plaque formation in AD brains (Aksenov et al., 1997; Robinson, 2000).

Besides the effect of ammonia on glutamatergic tone, ammonia also could alter the gamma-aminobutyric acid (GABA) system in the brain. GABA is one of the factors that mediate inhibitory neurotransmission. For example, excessive levels of ammonia increase GABA release, which leads to the enhancement of the GABAergic system in AD. Thus, neurotransmission imbalances caused by ammonia might be responsible for cognitive deficits in AD (Seiler, 2002; Rama Rao et al., 2010). However, the mechanisms by which ammonia contributes to the manifestations of AD remain poorly defined.

## Ammonia triggered inflammatory responses in AD

Elevated brain ammonia is capable of affecting crucial inflammatory processes causing alterations in the release of cytokines and inflammatory proteins by microglia, astroglioma, astrocytes, and neurons (Figure 2). Additionally, increased levels of ammonia induce apoptosis, which is associated with neuronal degeneration, via different signaling molecules such as nuclear factor-kappa B (NF-κB) (Buzanska et al., 2000). NF-kB is a transcription factor with the critical role in the regulation of inflammatory responses, innate immunity, apopotosis, and mitochondrial dysfunction (Barnes and Adcock, 1997; Henderson et al., 2002; Sinke et al., 2008; Shi et al., 2014). These include inducible nitric oxide synthase (iNOS), nitric oxide (NO), NADPH oxidase (NOX), superoxide and peroxynitrite, phospholipase A2 (PLA2), and cycooxygenase-2 (COX-2), which have been proven capable of inducing astrocyte swelling. Recent studies have also shown that ammonia activates iNOS, NOX, PLA2, and COX-2, and inhibition of aftermentioned enzymes significantly diminishes astrocyte swelling induced by ammonia. Additionally, immunohistochemical analysis has shown that ammonia-treated astrocyte cultures are able to increase NF-κB activity and astrocyte swelling. Blockage of NF-κB activity by BAY 11-7082, results in a reduction of ammonia-induced swelling in cultured astrocytes (Sinke et al., 2008; Rama Rao et al., 2010; Rao et al., 2010). These findings indicate a critical role of ammonia in activating NF-κB and ultimately astrocyte swelling. However, the mechanisms that underlie how NF-κB signaling pathways contribute to astrocyte swelling are not completely understood. This lack of understanding offers potential avenues for further research.

Evidence indicates that activation of inflammatory responses is a pathological hallmark of AD. Neuroinflammation in AD is marked by increased activity of inflammatory cytokines such as IL-6, IL-1β, and TNF-α. Because neuroinflammation is one of key factors in both AD and the hyperammonia condition, it is hypothesized that targeting neuroinflammatory mediators such as NF-κB could provide an effective strategy for the treatment of neurological abnormalities associated with elevated ammonia levels.

## Ammonia and memory

Memory disruption is one of the major neuropathological hallmarks in AD (Albert, 1996). The elevation of ammonia concentrations progressively leads to impaired mental status (cognitive, spatial learning, and memory dysfunctions). In 2000, Aguilar et al. showed that exposure of rat hippocampal slices to high ammonia concentrations compromised NMDA receptors, which subsequently impairs memory or conditioned learning in the animals (Aguilar et al., 2000). Other possible mechanisms of the learning deficits produced by high levels of ammonia most likely involve a reduction of the neuronal glutamate-nitric oxide (NO)-cyclic GMP pathway. Interestingly, reduction of NO formation correlated with intellectual dysfunction in AD dementia patients, but not in those with vascular dementia (Kosenko et al., 2014).

In addition, it has been suggested that chronic ammonia exposure affects cognitive function through neurosteroid metabolism. Ammonia impairs the synthesis of neurosteroids, which is believed to be involved in memory impairment. Also, it has been shown that ammonia impairs long-term potentiation (LTP) in the hippocampus, which a mechanism is thought critically involved in memory formation. Ultimately, ammonia may modulate neurosteroid metabolism via excessive activation of NMDA receptors and the inhibition of LTP through a GABA receptor-mediated effect (Izumi et al., 2013).

## Conclusion

Just over 20 years have passed since the first hypothesis was published suggesting there might be a link between ammonia and AD (Seiler, 1993). Since then, very few direct investigations have been in favor of a pathophysiological role for ammonia in AD brain. However, since high brain ammonia concentrations have been detected in AD, characterization of physiological and molecular mechanisms of the ammonia transport system in brain cells of AD vs. control will now yield a better understanding of whether ammonia transport is altered in AD. Gaining knowledge on nitrogen transport mechanisms and its regulation in AD will have direct relevance to the medical field.

Although, ammonia is not likely a primary cause of AD, it acts as a potent neurotoxin affects various biological pathways such as impairment of energy metabolism, mitochondrial dysfunction, dysregulation of inflammatory response, and memory dysfunction. Interestingly, these pathways also contribute to the generation and/or progression of AD. Aforementioned factors have also been observed in AD. Thus, more research is required to investigate the potential linkage between ammonia toxicity and AD. It is believe that investigating this potential linkage will greatly assist in developing therapeutic drugs for modifying dangerous ammonia influxes in AD brain and the prevention of lethal brain cell damage in AD.

## Author contributions

AA: formulated the study and wrote the manuscript. MS: designed figures. BA: provided intellectual thoughts, revised the manuscript and project leader.

## Funding

This work was funded by Research Manitoba/Alzheimer's Society and St. Boniface Hospital Research Foundation grants [grant numbers: 1406-3216, 1403-3131, and 1410-3216].

### Conflict of interest statement

The authors declare that the research was conducted in the absence of any commercial or financial relationships that could be construed as a potential conflict of interest.
